# Low 25-hydroxyvitamin D concentrations in wild rabbits (*Oryctolagus cuniculus*) in southern Finland

**DOI:** 10.1186/s13028-024-00726-0

**Published:** 2024-02-06

**Authors:** Johanna Mäkitaipale, Pinja Hietanen, Thomas Grönthal

**Affiliations:** 1 Department of Equine and Small Animal Medicine, Faculty of Veterinary Medicine, University of Helsinki, P.O. Box 57, FI-00014 HelsinkiUniversity of Helsinki, Finland; 2 Finnish Food Authority, P.O. Box 100, FI-00027 Finnish Food Authority, Finland

**Keywords:** Endogenous synthesis, Hypovitaminosis D, Vitamin D deficiency, Vitamin D synthesis

## Abstract

**Background:**

Diet and endogenous vitamin D synthesis are possible sources of vitamin D in wild rabbits. Higher 25-hydroxyvitamin D (25(OH)D) concentrations have been reported in rabbits after artificial UVB light exposure than in rabbits without this exposure, suggesting that endogenous vitamin D synthesis occurs in the former group. In Finnish pet rabbits, diet was reported as main source of vitamin D, while outdoor access was not. Finland’s northern location only enables endogenous synthesis from mid-March to mid-October in people with light skin type. Living conditions during winter are challenging for Finnish wild rabbits. This study aimed to measure serum 25(OH)D concentrations and possible natural seasonal variation of vitamin D concentrations in Finnish wild rabbits.

**Results:**

Post-mortem blood samples (n = 78) were collected between 2013 and 2021 from wild rabbits hunted for reduction of the wild rabbit population. Separated sera were stored at − 80 °C until 25(OH)D concentrations were measured by enzyme immunoassay. Data regarding sex were available from 50 rabbits, 29 (58%) of which were females. Mean 25(OH)D concentration was 3.3 (range 0.3–7.1) ng/ml. 25(OH)D concentration was statistically similar between season (autumn, winter, summer), month or year of sample collection, and sex.

**Conclusions:**

Wild rabbits living in Finland have very low serum 25(OH)D concentrations. This is far below the previously suggested threshold of vitamin D deficiency in rabbits (17 ng/mL) or the mean 25(OH)D concentration reported in Finnish pet rabbits (26.0 ng/mL). Seasonal variation was not observed in 25(OH)D concentrations between winter and summer months. Even though rabbits are crepuscular animals and may spend the mid-day in underground burrows, the very low observed 25(OH)D concentrations raise doubt about whether vitamin D synthesis occurs efficiently in the skin of rabbits and whether the diet of wild rabbits provides adequate amounts of vitamin D. Cutaneous vitamin D synthesis, possible long-term consequences of low 25(OH)D concentrations, and the association of low vitamin D status with other health disorders warrant further investigations in rabbits.

## Background

Vitamin D is a secosteroid hormone, the main function of which is the maintenance of calcium balance. Rickets in children and osteomalacia in adults are consequences of severe vitamin D deficiency and these are well-recognized also in rabbits fed a vitamin D-deficient diet [1–5]. Vitamin D is also important in many other metabolic functions, including growth, immune response, and neuromuscular activity, and deficiency has been linked to numerous common health disorders in humans [4, 6]. These have been much less studied in rabbits.

Diet, vitamin D supplements, and endogenous vitamin D synthesis in skin after sun exposure are possible sources of vitamin D in domestic rabbits [1, 7, 8]. Plants at a later stage of maturity may contain vitamin D_2_ (ergocalciferol). Ergocalciferol is synthesized from plant-based sterol ergosterol after sun exposure in the cell membranes of endophytic fungi that contaminate plant material such as hay [9]. Leaves of some plants mainly belonging to the Solanaceae family contain vitamin D_3,_ (cholecalciferol) derived from sterol 7-dehydrocholesterol after sun exposure [9]. Commercial rabbit food fortified with vitamin D_3_ serves as a source of vitamin D for domestic rabbits. Vitamin D supplements are not generally recommended for rabbits due to the risk of vitamin D overdose [10].

Exposure to artificial UVB light has been shown to increase serum 25-hydroxyvitamin D concentration in rabbits, suggesting the existence of endogenous vitamin D synthesis in this species [7, 8]. During exposure to sunlight or artificial UVB light, radiation penetrates the skin and converts 7-dehydrocholesterol (provitamin D_3_) to previtamin D_3_, and further to vitamin D_3_, which is transported to the liver. Vitamin D_2_, obtained from the diet, and vitamin D_3_, obtained from endogenous synthesis, are converted in the liver to 25-hydroxyvitamin D_2_ (25(OH)D_2_) and 25-hydroxyvitamin D_3_ (25(OH)D_3_), respectively. These together form 25-hydroxyvitamin D (25(OH)D), which is most commonly used to determine vitamin D status from blood samples.

A recent study reported that one-third of pet rabbits had serum 25(OH)D below the suggested vitamin D deficiency threshold of 17 ng/ml [11]. The main vitamin D source of pet rabbits was the diet, and outdoor access was not associated with the 25(OH)D concentrations [12]. The aim of the current study was to measure serum 25(OH)D concentrations in Finnish wild rabbits whose diet and outdoor access are unaffected by human intervention. This would yield important information regarding the natural seasonal variation of 25(OH)D concentrations in rabbits, which would be beneficial in the planning of pet rabbits’ living conditions and outdoor access recommendations. We hypothesized that serum 25(OH)D concentrations are higher in wild rabbits during late summer and autumn months than during winter months.

## Methods

### Serum samples

This cross-sectional study aimed to measure serum 25(OH)D concentrations and possible natural seasonal variation of vitamin D concentrations in Finnish wild rabbits. Blood samples were collected from wild rabbits hunted from urban Helsinki city area by hunters as part of a yearly project of culling the wild rabbit population. The aim was to get samples throughout the hunting season from September to March during 2013, 2019, 2020, and 2021. Rabbits were hunted in accordance with the Finnish Hunting Act and Hunting Decree and for other reasons than this study. After death, blood samples were obtained either by cutting the *vena jugularis* and *arteria carotis* and dripping directly into serum tubes or by aspirating the sample with a 5 mL syringe and 18-gauge needle from the heart or abdominal aorta. Blood samples were centrifuged at 1485 × g (4000 rpm). Serum was collected and frozen at − 80 °C until the 25(OH)D concentrations were determined. An enzyme immunoassay (25-hydroxyvitamin D EIA kit AC-57SF1, Immunodiagnostic Systems Holdings PLC, Tyne & Wear, UK) was performed according to the manufacturer’s instructions and as previously described [12].

### Statistical analysis

Data analysis and statistics were carried out using IBM SPSS Statistics (v. 27, IBM Statistics, New York, NY, USA). Normality of data distribution was assessed by Kolmogorov–Smirnov test for serum 25(OH)D concentration. Mann–Whitney U test was used for analyses with two variables and Kruskal–Wallis H test for analyses with more than two variables. Months of sample collections were categorized as winter (December, January, February), spring (March, April, May), and autumn (September, October, November) for further analyses. The impact of the season on UVB-induced vitamin D synthesis was assessed by grouping samples into those collected between March and October and those collected between November and February. P values < 0.05 were considered statistically significant.

## Results

**Fig. 1:**
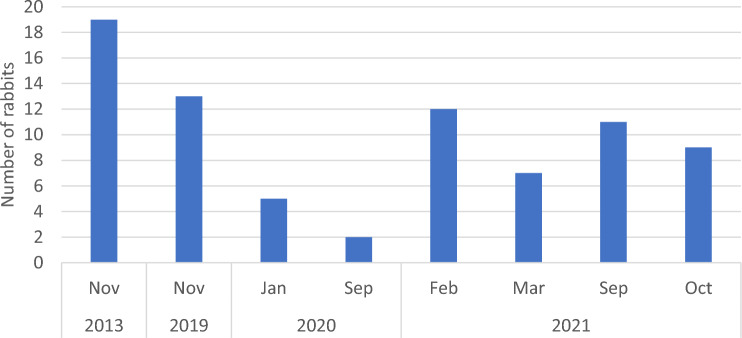
Number of wild rabbit specimens for 25-hydroxyvitamin D measurement per month and year

In total, 78 wild rabbit serum samples were collected between November 2013 and October 2021 (Fig. 1). 25(OH)D concentrations were measurable in 64 of these samples. In 14 samples, the concentration was below the limit of measurement. Data regarding sex were available for 50 rabbits, of which 29 (58%) were females and 21 (42%) were males (Table 1). Mean 25(OH)D concentration was 3.3 (range 0.3–7.1) ng/mL. Concentration was similar in females and males (U = 121.5, z = − 1.407, P = 0.161, median 3.7 ng/mL and 3.0 ng/mL, respectively). The month of sample collection did not affect 25(OH)D concentration (H(5) = 3.002, P = 0.700). 25(OH)D concentrations were similar between samples collected during winter, spring, and autumn (H(2) = 0.903, P = 0.637), in both females (H(2) = 5.178, P = 0.075) and males (H(2) = 2.493, P = 0.288). Year of sample collection did not affect 25(OH)D concentration (H(3) = 1.369, P = 0.713). No differences were observed in samples collected between March and October and between November and February (U = 410.5, z = − 1.265, P = 0.206).

**Table 1:** Results of 25-hydroxyvitamin D concentrations in wild rabbit serum specimens taken in Finland during 2013–2021

Variable	n^1^	25(OH)D-vitamin average (ng/mL)	95% Confidence interval (ng/mL)	P value (statics)
Sex	Female: 29 Male: 20 Unknown: 31	3.8 2.9	3.0–4.6 1.9–3.9	0.16 (U = 121.5 z = − 1.4)
Month of sampling	September: 13 October: 9 November: 33 January: 5 February: 11 March: 9	3.8 3.3 3.0 3.9 2.9 3.5	2.6–5.1 2.1–4.7 2.4–3.7 0.1–7.8 1.9–3.9 2.0–5.2	0.70 (H(5) = 3.00)
Year of sampling	2013: 19 2019:14 2020: 9 2021: 38	3.0 3.1 3.8 3.4	2.4–3.6 0.0–9.3 1.8–5.8 2.9–4.0	0.71 (H(3) = 1.369)
Season of sampling^**2**^	Winter: 16 Spring: 9 Autumn: 55	All: 3.1 Females: 3.0 Males: 3.2 All: 3.8 Females: 3.5 Males: 4.6 All: 3.35 Females: 5.06 Males: 2.40	2.1–4.1 1.9–4.1 0.0–7.1 2.4–5.2 1.5–5.4 1.1–8.0 2.8–3.9 3.5–6.6 1.2–3.6	0.63 (H(2) = 0.90) 0.07 (H(2) = 5.178) 0.28 (H(2) = 2.493)
Age group	Young: 3 Adult: 35 Unknown: 42	3.7 3.3	0.0–12.0 2.6–4.0	0.64 (U = 20.0 z = − 0.643)
Vitamin D synthesis season	March-October: 31 November-February: 49	3.7 3.1	3.0–4.4 2.6–3.6	0.20 (U = 410.5. z = − 1.265)

^1^ Number of rabbits

^2^ Winter = December, January, February

Spring = March, April, May

Summer = June, July, August

Autumn = September, October, November

## Discussion

Wild rabbits living in Finland located at 60 ºN have very low serum 25-hydroxyvitamin D concentrations, as mean concentration was only 3.3 ng/mL. Season of sample collection did not affect concentrations despite UVB radiation being higher during summer months. By contrast, using the same analysis method, a mean 25(OH)D concentration of 26.0 ng/mL was reported in Finnish pet rabbits [12]. Outdoor access during summer months was not associated with 25(OH)D concentration, and diet was determined to be the main source of vitamin D in pet rabbits [12]. The diet of wild rabbits consists of fresh grass and plants during summer and is very restricted during winter when snow covers the ground. As ergocalciferol is synthesized after sun exposure in the cell membrane of fungi- contaminated plant material, fresh grass and plants have low vitamin D content. The diet of Finnish wild rabbits during winter comprises mainly twigs, roots, bark, and conifer needles [13]. In urban areas, wild rabbits also eat flowers from cemeteries and seeds from bird feeders [13]. These are all poor sources for vitamin D and cannot compensate for the lack of endogenous vitamin D synthesis during winter months. In pet rabbits’ diet, good-quality dry hay in Finland contains vitamin D precursors at approximately 1000 IU/kg and commercial rabbit, horse, and cattle food fed to Finnish pet rabbits at 1000− 3000 IU/kg [12]. The differences in dietary vitamin D levels therefore likely explain the differences in 25(OH)D concentrations between wild and pet rabbits.

Living conditions for wild rabbits in Finland are challenging and differ from those of the Iberian Peninsula, where rabbits originate [14]. Endogenous vitamin D synthesis in the skin is possible in Southern Finland for people with light skin types (I–III) only between mid-March and mid-October if the exposure time is at least 30 min [15]. For people with darker skin types (IV–VI), synthesis occurs only during one or two summer months [15]. Rabbits are crepuscular animals and might therefore spend mid-day in underground burrows. Mid-day is the best time for endogenous vitamin D synthesis, and wild rabbits may therefore miss the opportunity for endogenous vitamin D synthesis, as was suspected to happen in pet rabbits with limited outdoor access [12]. In the Iberian Peninsula, located at latitude 40 ºN, where UVB radiation is higher than in Finland, endogenous vitamin D synthesis is possible throughout the year [15]. People with light skin types (I–III) can receive one standard vitamin D dose (SDD) when 1/4 of the body surface is exposed for 15 min during 9.5 months of the year [15]. This corresponds to an oral dose of about 1000 IU (25 µg) of vitamin D [15]. If exposure time is elongated to 60 min, endogenous vitamin D synthesis is possible at latitude 40 ºN throughout the year [15].

Previously, 25(OH)D concentration of 17 ng/mL was suggested as a threshold for vitamin D deficiency in rabbits [11]. Using this benchmark, all wild rabbits in our study appeared to have severe vitamin D deficiency. In humans, the threshold for severe vitamin D deficiency is 12 ng/mL, but concentrations of up to 40 ng/ml are recommended for optimal cellular health [16, 17]. The lifespan of wild rabbits is short relative to pet rabbits, with rabbits in the wild rarely reaching the age of 3 years [18]. Life expectancy of a newborn rabbit is 70 days, and 40% of adult rabbits die before beginning the second reproductive season [18]. The most common reasons for mortality are diarrhoea due to coccidiosis [18] and rabbit haemorrhagic disease, other diseases, predators, and accidents [19]. The possible association of vitamin D deficiency in these diseases has not been investigated. In humans, optimal vitamin D status has been recognized as a front-line factor in prophylaxis for musculoskeletal disorders, infections and autoimmune diseases, cardiovascular disease, type 1 and 2 diabetes mellitus, several types of cancers, neurocognitive dysfunction and mental illness, and reproductive diseases [20].

Calcium metabolism in rabbits differs from that of many other mammals. Calcium absorption from the intestines is passive [2]. Vitamin D-dependent absorption is needed only if dietary calcium content is low. Almost all dietary calcium is absorbed and blood calcium concentration in rabbits is therefore higher than in other mammals. Calcium metabolism has similarities with that of horses. In horses, low serum 25(OH)D concentrations have also been reported [21]. When 25(OH)D concentrations were compared in pasturing horses with or without full-covering blankets, no differences were observed [21]. Serum 25(OH)D_2_ concentrations were, however, higher than 25(OH)D_3_ concentrations during the summer months in both groups [21]. The authors postulated that horses were dependent on dietary sources of vitamin D instead of endogenous synthesis [21]. In rabbits, higher 25(OH)D concentrations were observed after artificial UVB light exposure [7, 8]. These studies measured the total 25(OH)D concentration, as did ours. Measuring 25(OH)D_2_ and 25(OH)D_3_ separately would likely yield more information regarding the source of the vitamin D. In an artificial UVB light study, rabbits were fed commercial rabbit food and timothy hay [7, 8]. Vitamin D in hay originates from high amount of ergosterol in cell membranes of endophytic fungi that is synthesised to D_2_ after UVB exposure. As the 25(OH)D_2_ and 25(OH)D_3_ concentrations were not measured separately in the previously mentioned artificial UVB study, it is possible that ergocalciferol level in the hay increased during the trial, explaining the increase in serum total 25(OH)D concentrations.

Our study has some limitations. The lack of separate analyses of 25(OH)D_2_ and 25(OH)D_3_ concentrations is a clear drawback. The need for separate analyses was noted only after receiving the unexpectedly low results. In addition, blood sample collection was spread over several years due to difficulties in sample collection. The rabbits were mainly hunted at night in different locations, making sample collection logistically challenging. Also, rabbit haemorrhagic disease and myxomatosis spread in Finland during this period, reducing the wild rabbit population radically. Some samples were thus frozen for several years. However, 25(OH)D has been shown to be very stable, up to 24 years when stored at − 24 ºC [22]. A decrease of only 2.3% in 25(OH)D concentration was observed after human whole-blood samples were stored for 72 h at room temperature [23]. In our study, samples were collected as soon after death as possible. As capture was done mainly during colder months, temperature was unlikely to be a significant factor. Some of the samples were haemolytic and some lipaemic. According to the manufacturer, haemoglobin up to 1470 mg/dL, bilirubin up to 513 µmol/L, and triglyceride up to 5.6 mmol/L do not interfere with the assay used, and thus, haemolysis and lipaemia were unlikely to affect the results. While no apparent diseases were observed among the wild rabbits caught in this study, it is possible that our sample may have been biased if rabbits with exceptionally low 25(OH)D concentrations were more likely to be caught.

## Conclusions

Our findings suggest that 25(OH)D concentrations among wild rabbits in southern Finland between September and March are very low. Further research is required to evaluate the effects of UVB light on serum 25(OH)D_3_ concentrations in rabbits. Also, the long-term health impact of vitamin D deficiency in rabbits warrants investigation.

## Data Availability

The datasets used and analysed during the current study are available from the corresponding author on reasonable request.
